# Dissecting the phenotypic heterogeneity in sensory features in autism spectrum disorder: a factor mixture modelling approach

**DOI:** 10.1186/s13229-020-00367-w

**Published:** 2020-08-31

**Authors:** J. Tillmann, M. Uljarevic, D. Crawley, G. Dumas, E. Loth, D. Murphy, J. Buitelaar, T. Charman, the AIMS-2-TRIALS LEAP group, Jumana Ahmad, Sara Ambrosino, Bonnie Auyeung, Sarah Baumeister, Christian Beckmann, Thomas Bourgeron, Carsten Bours, Michael Brammer, Daniel Brandeis, Claudia Brogna, Yvette de Bruijn, Bhismadev Chakrabarti, Ineke Cornelissen, Flavio Dell’ Acqua, Guillaume Dumas, Christine Ecker, Jessica Faulkner, Vincent Frouin, Pilar Garcés, David Goyard, Hannah Hayward, Joerg Hipp, Mark H. Johnson, Emily J. H. Jones, Prantik Kundu, Meng-Chuan Lai, Xavier Liogier D’ardhuy, Michael Lombardo, David J. Lythgoe, René Mandl, Luke Mason, Andreas Meyer-Lindenberg, Carolin Moessnang, Nico Mueller, Laurence O’Dwyer, Marianne Oldehinkel, Bob Oranje, Gahan Pandina, Antonio M. Persico, Barbara Ruggeri, Amber Ruigrok, Jessica Sabet, Roberto Sacco, Roberto Toro, Heike Tost, Jack Waldman, Steve C. R. Williams, Caroline Wooldridge, Marcel P. Zwiers

**Affiliations:** 1 Department of Psychology, Institute of Psychiatry, Psychology & Neuroscience, King’s College London, De Crespigny Park, Denmark Hill, London, SE5 8AF UK; 2 Department of Applied Psychology: Health, Development, Enhancement, and Intervention, University of Vienna, Vienna, Austria; 3 Division of Child and Adolescent Psychiatry, Department of Psychiatry and Behavioral Sciences, School of Medicine, Stanford Autism Center, Stanford University, Stanford, CA USA; 4 Melbourne School of Psychological Sciences, Faculty of Medicine, Dentistry, and Health Sciences, University of Melbourne, Melbourne, VIC Australia; 5 School of Psychological Science, Olga Tennison Autism Research Centre, La Trobe University, Melbourne, VIC 3086 Australia; 6 Department of Forensic and Neurodevelopmental Sciences, Institute of Psychiatry, Psychology and Neuroscience, King’s College London, London, UK; 7 Human Genetics and Cognitive Functions, Institut Pasteur, UMR3571 CNRS, Université de Paris, Paris, France; 8 Sackler Institute for Translational Neurodevelopment, Institute of Psychiatry, Psychology and Neuroscience, King’s College London, London, UK; 9 South London and Maudsley NHS Foundation Trust (SLaM), London, UK; 10 Department of Cognitive Neuroscience, Radboud University Medical Center, Nijmegen, The Netherlands; 11 Donders Institute for Brain, Cognition and Behavior, Radboud University, Nijmegen, The Netherlands; 12 Karakter Child and Adolescent Psychiatry University Centre, Nijmegen, The Netherlands

**Keywords:** Autism spectrum disorder, Phenotype, Sensory features, Heterogeneity, Social-communication symptoms, Anxiety

## Abstract

**Background:**

Heterogeneity in the phenotypic presentation of autism spectrum disorder (ASD) is apparent in the profile and the severity of sensory features. Here, we applied factor mixture modelling (FMM) to test a multidimensional factor model of sensory processing in ASD. We aimed to identify homogeneous sensory subgroups in ASD that differ intrinsically in their severity along continuous factor scores. We also investigated sensory subgroups in relation to clinical variables: sex, age, IQ, social-communication symptoms, restricted and repetitive behaviours, adaptive functioning and symptoms of anxiety and attention-deficit/hyperactivity disorder.

**Methods:**

Three hundred thirty-two children and adults with ASD between the ages of 6 and 30 years with IQs varying between 40 and 148 were included. First, three different confirmatory factor models were fit to the 38 items of the Short Sensory Profile (SSP). Then, latent class models (with two-to-six subgroups) were evaluated. The best performing factor model, the 7-factor structure, was subsequently used in two FMMs that varied in the number of subgroups: a two-subgroup, seven-factor model and a three-subgroup and seven-factor model.

**Results:**

The ‘three-subgroup/seven-factor’ FMM was superior to all other models based on different fit criteria. Identified subgroups differed in sensory severity from severe, moderate to low. Accounting for the potential confounding effects of age and IQ, participants in these sensory subgroups had different levels of social-communicative symptoms, restricted and repetitive behaviours, adaptive functioning skills and symptoms of inattention and anxiety.

**Limitations:**

Results were derived using a single parent-report measure of sensory features, the SSP, which limits the generalisability of findings.

**Conclusion:**

Sensory features can be best described by three homogeneous sensory subgroups that differ in sensory severity gradients along seven continuous factor scores. Identified sensory subgroups were further differentiated by the severity of core and co-occurring symptoms, and level of adaptive functioning, providing novel evidence on the associated clinical correlates of sensory subgroups. These sensory subgroups provide a platform to further interrogate the neurobiological and genetic correlates of altered sensory processing in ASD.

## Background

Notable heterogeneity in the configuration, severity, trajectory and treatment response of both core diagnostic and co-occurring psychiatric and behavioural symptoms in individuals with autism spectrum disorder (ASD) is well established [1, 2]. Lack of insight into the sources contributing to this heterogeneity has hampered progress towards understanding etiological mechanisms, developing effective treatments and predicting outcomes [3, 4]. We [3] and others [5] have suggested that a more fine-grained understanding of atypical sensory features may offer a promising approach to parse heterogeneity in ASD. The term ‘sensory features’ is used here to describe the diverse range of sensory symptoms in individuals with ASD that can encompass hyper-reactivity, hypo-reactivity and unusual sensory interests [6]. Indeed, a range of studies have linked atypical sensory features with restricted and repetitive behaviours [7, 8], socio-communicative impairments [9, 10], anxiety [11, 12], behavioural and sleep problems [13, 14] and adaptive functioning [15]. Further, several studies have identified the existence of potentially informative sensory-based subgroups among individuals with ASD [12, 16–19]. However, statistical and methodological limitations have precluded the field from fully capitalising on the potential utility of sensory features for explaining the heterogeneity of ASD [3].

Within the broader field of psychopathology, different methodological approaches have been put forward to investigate phenotypic heterogeneity [20]. Taxometric methods aim to address the question whether individual differences should be conceived as continuous traits (i.e. participants differ in degree of an observed behaviour) or in terms of typologies (i.e. participants belong to either of two qualitatively different types or latent subgroups [21];). While the former has been addressed using various methods of factor analysis (FA), the latter has been tested using cluster analysis and latent class analysis (LCA). In FA, latent factors capture the common content among test items and variability between participants is assumed to arise because of inter-individual differences on these factor variables. The latent factor(s) can be thus construed as a dimensional quantity upon which individuals *differ in degree.* Conversely, cluster analysis or LCA adopts a categorical view to explain heterogeneity: categorical latent classes or subgroups are assumed to capture variability between participants and individuals are classified based on their similarities in response pattern on a set of item variables. The term subgroup will be used throughout to refer to latent classes or clusters. In cluster analysis or LCA, variation between individuals is therefore assumed to relate to a *difference in kind*, and derived subgroups may differ qualitatively (i.e. subgroups present with a qualitatively different profile) or quantitatively (i.e. a high- or low-scoring subgroup).

To date, these two methodological approaches have been separately applied to investigate variability in sensory features in ASD. A limited but growing number of studies have used FA to delineate the underlying structure of sensory features as measured by commonly used parent-report questionnaire measures, including the Short Sensory Profile (SSP [22, 23]), Sensory Behaviour Questionnaire (SBQ [24]) and Sensory Experiences Questionnaire Version 3.0 (SEQ-3.0 [25]). Of these measures, the SSP [26] is one of the most widely used parent-report/caregiver questionnaire measure of sensory features in ASD [27] and has been used in large multicentre collaborative projects such as the Autism Speaks Autism Treatment Network [28] and the EU-AIMS Longitudinal European Autism Project [29]. The few existing studies that have applied FA to investigate sensory features in individuals with ASD as measured by the SSP have however produced inconclusive results, suggesting either a six- [22] or nine-factor structure [23] that only partially resembled the originally proposed seven-factor structure [26]. Reasons for these inconsistencies may relate to differences in sample size and age compositions, as well as the use of different FA techniques of varying specifications. In addition, some of the newly hypothesised constructs featured too few items to be psychometrically or clinically useful [23]. This suggests that it is currently not clear what the exact structure/taxonomy of sensory features in ASD is, which also limits previous studies that have utilised the SSP in subgrouping approaches.

In parallel to the above work, several studies have attempted to characterise heterogeneity in sensory features by identifying more homogeneous groups of individuals via different types of cluster analyses and latent class analyses (LCA) approaches (for a review see [30]). To date, studies have proposed anywhere from two to five subgroups using a range of different measures including the SSP [12, 18, 31], Sensory Experiences Questionnaire (SEQ [16]), Adolescent/Adult Sensory Profile (AASP [32]), Sensory Profile (SP [33]), Infant Toddler Sensory Profile (ITSP [34]) and Sensory Profile 2 (SP-2 [35, 36]). These instruments differ widely in the type of informant (i.e. self- vs. proxy-based), intended target population use (i.e. infants, children or adolescents/adults), sensory domains assessed and their psychometric properties [27, 37]. In addition, most studies have been limited by sample sizes and did not consider multiple developmental and clinical variables, leaving unanswered questions about the clinical correlates of sensory subgroups. Thus, it is not surprising that existing studies lack a clear consensus on the number of purported sensory subgroups in ASD, their frequency and profile, as well as associated clinical and demographic correlates. Despite some of these differences, two sensory subgroups were consistently identified: those with predominantly mild sensory features (i.e. referred to as ‘sensory adaptive’ or ‘perceptive-adaptable’) and those with marked impairments across all or most of sensory domains (i.e. termed ‘Sensory severe’, ‘Generalized sensory difference’, or ‘Sensorimotor’). Relevant to the current study, research using the SSP has identified either three sensory subgroups that differ in their severity of anxiety symptoms, but not on age, expressive language or social-communicative symptoms associated with ASD [12], or four sensory subgroups. The former was found in one study to differentiate in terms of age and level of adaptive behaviour [31], and in another study in age and non-verbal IQ, but not gender or ASD symptoms [18]. At least some of these inconsistencies are likely to be related to the varied choice of sensory measures employed across studies, as well as the different age and size of the sample studied [30].

While both FA and LCA approaches have been useful to further characterise sensory features in ASD, these taxometric procedures presuppose that sensory atypicalities either fall exclusively along a continuum from mild to severe or that individuals can be categorised into a finite number of discrete homogeneous entities or subgroups. Thus, the major limitation of FA is that it does not allow to classify individuals into groups, which is critical both in terms of informing clinical decision-making, but also for advancing neurobiological and genomic research and precision medicine approaches in ASD [38]. The major limitation of LCA and the categorical approach more broadly is that subgroups do not consider the range in severity and impairment within and across classes. Factor mixture modelling (FMM [39]) is a flexible hybrid model that combines LCA and FA approaches by simultaneously modelling the underlying structure to be both categorical and dimensional. The structure is considered categorical since the model allows for stratification of individuals into discrete subgroups while allowing for heterogeneity in the severity of the underlying trait within these groups through the use of continuous latent variables. This approach is particularly useful since it does not have the limitations of the two conventional taxometric procedures and it allows to directly compare different models of symptom structures. Indeed, FMM has been successfully applied to assess core diagnostic symptom structures in attention-deficit/hyperactivity disorder (ADHD [40, 41]) and ASD [42–45]. However, previous efforts in ASD focussed either on the two symptom dimensions of social communication and interaction and restricted and repetitive behaviours (RRB [42–44, 46]) or on empathy and systemising [45].

Despite the utility of this approach, FMM has not been used to characterise sensory features in ASD. Therefore, our study sought, for the first time, to apply FMM to compare dimensional, categorical and dimensional-categorical hybrid structures of sensory features in a large and well-characterised sample of individuals with ASD. Our aim was to clarify whether the structure of sensory features in ASD can be best conceptualised either by (1) a continuum on which individuals differ in severity, (2) sensory subgroups that display either quantitative or qualitative differences in their sensory profiles or (3) a multidimensional factor model composed of sensory subgroups that differ in both their severity within and across groups along specific continuous factor scores. In addition, we aimed to further characterise identified groups in terms of potential differences in age, gender distribution and IQ, as well as how they relate to individual differences in social communication and RRB symptoms, co-occurring symptoms of anxiety and ADHD and adaptive functioning. With few exceptions, previous subgrouping studies have not simultaneously examined potential associated clinical variables. It remains therefore unclear how sensory subgroups also differ in other aspects of core ASD and co-occuring symptoms, as well as adaptive functioning.

## Methods

### Participants

The sample comprises 332 individuals with ASD ranging in age from 6 to 31 years (*M* = 16.9, SD = 5.95) recruited as part of a multisite longitudinal study (EU-AIMS LEAP [47]). All participants had an existing clinical diagnosis of ASD according to DSM-IV [48], DSM-IV-TR [49], DSM-5 [50] or ICD-10 [51] criteria. For further in-depth clinical information of the cohort, we refer to [47]. Descriptive statistics for the current sample are listed in Table 1. Informed consent was obtained for all participants in the study in accordance with the Declaration of Helsinki, and Institutional Research Ethics Boards at all sites approved the research procedures.

**Table 1:** Sample characteristics (*N* = 332)

	*N*	Mean	SD	Range
Sex (males:females)	237:95	–	–	–
Age in years	332	15.95	(5.63)	6–31
ADOS CSS-SA	323	6.46	(2.58)	1–10
ADOS CSS-RRB	323	4.85	(2.77)	1–10
SRS-2 SCI	320	73.76	(11.82)	42–102
VABS socialisation	285	69.44	(16.52)	20–119
VABS daily living	286	72.35	(16.95)	25–131
VABS communication	290	74.87	(17.14)	21–130
VABS ABC	281	70.07	(15.03)	20–121
Nonverbal IQ	326	97.15	(22.36)	44–150
Verbal IQ	322	95.44	(21.14)	45–160
Full-scale IQ	326	96.35	(20.87)	40–148

*SD* Standard deviation, *SSP* Short Sensory Profile, *ADOS CSS-SA-RRB* Autism Diagnostic Observation Schedule Calibrated Severity Scores for Social Affect and Restricted and Repetitive Behaviours, *SRS-2 SCI* Social Responsiveness Scale–2 Social Communication and Interaction total raw scores, *VABS ABC* VABS Adaptive Behaviour Composite, *IQ* intelligence quotient

VABS domain scores are standardised scores (age normalised: *M* = 100, SD = 15)

### Short Sensory Profile

The *Short Sensory Profile* (SSP [52]), a shortened version of the *Sensory Profile* (Dunn, 1999), is one of the most commonly used parent-report/caregiver questionnaire measure of sensory features in ASD [27]. The SSP is composed of 38 items that probe sensory processing in the context of daily activities. For each item, parents or caregivers report on a 5-point Likert scale: 1 = always, 2 = frequently, 3 = occasionally, 4 = seldom and 5 = never. Based on a normative sample of 1200 typically developing children and derived through EFA, the SSP measures sensory features in seven domains: tactile sensitivity, taste/smell sensitivity, movement sensitivity, underresponsive/seeks sensation, auditory filtering, low energy/weak and visual/auditory sensitivity [26]. A total score across the 38 items was obtained that reflects function across multiple sensory domains. For domain and total scores, lower scores indicate more sensory impairment.

### Subgroup correlates

Subgroup correlates were chosen based on their conceptual value of providing important insight into associations with demographic indicators (age, sex/gender, intellectual functioning), ASD-specific behaviours (e.g. social-communication symptoms and restricted and repetitive behaviours) and symptoms of anxiety and attention-deficit/hyperactivity disorder (ADHD).

The *Autism Diagnostic Observation Schedule* (ADOS-G [53], ADOS-2 [54]) is a clinician-administered instrument to assess social communication and interaction, stereotyped behaviours and restricted interests in a semi-structured observational setting. Calibrated severity score (CSS) for the core symptom domains of social communication (i.e. social affect) and restricted and repetitive behaviours (RRB) were derived from the ADOS-2 algorithm. The CSS ranges from 1 to 10, with higher scores indicating more severe ASD symptom severity. The *Social Responsiveness Scale*, *Second Edition* (SRS-2 [55]) is a dimensional measure of autistic traits comprising 65 items each rated on a 0 (‘not true’) to 3 (‘almost always true’) Likert scale. SRS-2 parent-reported total raw scores for social communication and interaction (SCI) were used to capture specifically autistic traits relating to social-communication deficits. The *Repetitive Behaviour Scale-Revised* (RBS-R [56]) probes for restricted and repetitive behaviours (RRBs) associated with ASD. Based on Lam and Aman [57], five subscales were derived to investigate specific RRBs: stereotyped behaviour, self-injurious behaviour, compulsive behaviour, ritualistic/sameness behaviour and restricted behaviour.

Co-occurring symptoms of anxiety were measured using the Development and Well-Being Assessment (DAWBA [58]), a semi-structured parent/carer interview that generates risk prediction scores according to ICD-10 [51] and DSM-IV-TR [49] criteria. DAWBA scores are distributed on an ordinal scale and reflect six levels of prediction of the probability of meeting clinically relevant diagnostic criteria for a disorder, ranging from very unlikely (~ 0.1%) to probable (risk score > 70%). Following previous studies, a pooled anxiety prediction score reflecting an individual’s highest risk score across a group of anxiety disorders (OCD, generalised anxiety, panic disorder, agoraphobia, PTSD, separation anxiety, social phobia and specific phobia) was created [59, 60].

Symptoms of ADHD were assessed with the DSM-5 rating scale that covers 18 items measuring the presence of inattention and hyperactive/impulsive symptoms, each evaluated by parents/caregivers on a 0–3 scale (0 = not at all to 3 = very often). The level of intellectual abilities was assessed with either the *Wechsler Abbreviated Scales of Intelligence-Second Edition* (WASI-II [61]) or if unavailable the *Wechsler Intelligence Scale for Children-III/IV* (WISC-III/IV [62, 63]) in children and *Wechsler Adult Intelligence Scale for Adults-III/IV* (WAIS-III/IV [64, 65]) in adults. Standardised estimates of verbal IQ (VIQ), performance IQ (PIQ) and full-scale IQ (FSIQ) with *M* = 100 and SD = ± 15 are reported.

Adaptive functioning was assessed using the Vineland Adaptive Behaviour Scale-Second Edition (VABS-II [66]), a semi-structured parent interview that measures adaptive functioning across three domains in > 6-year-olds: communication, socialisation and daily living skills. For each domain, standard scores were obtained and combined to generate an Adaptive Behaviour Composite (ABC) score. VABS standard scores have a mean of 100 (SD = 15), with lower scores indicating greater functional impairment.

### Statistical analysis

In the current study, we applied factor mixture modelling (FMM) to test a multidimensional factor model of sensory processing in ASD by identifying simultaneously more homogenous sensory subgroups in ASD that differ in their severity within and across groups along continuous factor scores. FMM integrates both confirmatory factor analysis (CFA) and latent class analysis (LCA) approaches to concurrently model continuous factors (i.e. dimensional trait variability) and categorical latent factors (i.e. subgroups) to explain heterogeneity [67]. More technically, FMM models fit of competing latent structural models that are composed of both categorical and continuous structures in a single analytical framework. Different FMMs (i.e. different competing structural models) can be compared by using well-established comparative indices of goodness-of-fit [39]. Maximum likelihood with robust standard errors (MLR) was used as the method of estimation, as it yields Akaike information criterion (AIC) and Bayesian information criterion (BIC) values that can be used to compare results across analyses approaches (LCA, CFA and FMM). Lower AIC and BIC values indicate better fit of the model, with the lowest value in a comparison indicating the best and most parsimonious fit of a model relative to all other specified models. A simulation study has shown that BIC performs better or equal compared to other fit indices including AIC and the adjusted BIC [68]. We therefore focus predominantly on BIC values when comparing different structural models. To interpret meaningfully differences between models, Raftery [69] suggested that a 10-point difference in BIC values provides very strong evidence (i.e. odds ratio = 150:1) that the model with the lowest BIC value is the better-fitting model. To further guide the decision on the number of classes in FMM models, two likelihood-ratio tests (Lo-Mendell-Rubin (LMR) and the bootstrap likelihood ratio test (BLRT) are also reported. These likelihood-ratio tests compare the improvement in fit between neighbouring class models (e.g. comparing *k-1* and the *k* class models). CFA, LCA and FMM were all run using the MPlus software version 6.12 [70].

To investigate differences between sensory subgroups in other clinical variables of interest, effect sizes (ES) were estimated that reflect mean differences between two groups divided by the total standard deviation of all groups combined. ES are presented as Cohen’s *d* with conventions of very small (*d* < 0.2), small (*d* = 0.2), medium (*d* = 0.5) and large (*d* ≥ 0.8). Subgroups were compared on the following clinical variables: sex, age, full-scale IQ, symptoms relating to social communication and interaction (SCI), restricted and repetitive behaviours (RRB), symptoms of anxiety and ADHD and adaptive functioning. To test the statistical significance of mean group differences across multiple dependent variables simultaneously, a multivariate multiple regression analysis was conducted. Group comparisons factored in the effects of age and full-scale IQ on the dependent variables to test the unique effect of sensory class on clinical variables. Age, IQ, ASD symptoms, symptoms of ADHD and adaptive functioning were entered as continuous predictors. Since symptoms of anxiety were measured on an ordinal scale (DAWBA risk bands of 0–5), polynomial contrasts (linear, quadratic and cubic effects) were fit. Subgroup 3, the sensory low group, was chosen as reference group for all comparisons. To increase confidence in the robustness of the results obtained, an α-level of < 0.01 was applied for all statistical analyses. Descriptive analyses (effect size differences between classes) and analyses relating to group correlates were conducted using the STATA software 15.0 [71].

## Results

The 38 items of the SSP measuring sensory features were first subjected to a series of CFAs with correlated factors to evaluate model fit of three models specified a priori: the original 7-factor solution [26], a 6-factor solution [22] and a novel 5-factor solution that has been partially proposed in previous studies [8, 72], but has never been formally tested in terms of its psychometric properties. A detailed description of all factor models tested can be found in the Supplementary Materials.

Results indicated that the 5-factor model provided the poorest fit, as AIC and BIC values were highest for this model. The 6-factor model as suggested by Tomchek and colleagues [22] provided the next best fit to the data followed by the 7-factor model, which provided the best fit to the data, as it yielded the lowest AIC and BIC values—BIC values were 295 to 387 points lower than the other models (see Table 2). To account for the ordinal nature of the SSP item data (i.e. scores from 1–5) and to report the least-biased measures of model fit, all CFA analyses were re-run using mean- and variance-adjusted weighted least squares (WLSMV) estimation method [73]. Results using WLSMV replicated the findings using MLR and identified the 7-factor solution as the most parsimonious continuous factor solution (see Supplementary Materials for a detailed summary of results). The best performing CFA factor solution, the 7-factor structure, was subsequently used in two FMMs that varied in the number of subgroups. Specifically, we tested a two-subgroup, seven-factor model and a three-subgroup and seven-factor model. To confirm whether FMMs provide a better overall fit to the data than the subgroup models proposed in previous studies, four different LCA models (with two-to-six subgroups) based on participant’s item response patterns were also evaluated.

**Table 2:** Comparison of different structural models of sensory symptoms in ASD, fit indices and subgroup proportions (*N* = 332)

Model	Log-likelihood	Par	AIC	BIC	Adjusted BIC	LMR *p* value	BLRT *p* value	Class percentages
Latent class analysis								
Two-subgroup	− 19,313	115	38,856	39,294	38,929	.004	< .0001	55%, 45%
Three-subgroup	− 18,812	154	37,932	38,518	38,029	.024	< .0001	27%, 33%, 40%
Four-subgroup	− 18,571	193	37,527	38,262	37,650	.141	< .0001	17%, 30%, 15%, 37%
Five-subgroup	− 18,359	232	37,182	38,064	37,329	.160	a	17%, 14%, 34%, 19%, 16%
Six-subgroup	− 18,245	271	37,032	38,063	37,204	.745	a	17%, 10%, 15%, 21%, 22%, 15%
Factor analysis								
Five-factor	− 17,623	124	35,495	35,967	35,574	–	–	
Six-factor	− 17,563	129	35,384	35,875	35,466	–	–	
Seven-factor	− 17,398	135	35,066	35,580	35,152	–	–	
Factor-mixture analysis								
Two-subgroup, seven-factor	− 17,362	142	35,009	35,549	35,099	.0021	< .0001	19%, 81%
**Three-subgroup, seven-factor**	− 17,301	150	34,902	35,473	34,997	.01	a	7%, 15%, 77%
Four-subgroup, seven-factor	b							

*Par* number of estimated parameters, *AIC* Akaike information criterion, *BIC* Bayesian information criterion, *LMR* Lo-Mendell-Rubin test, *BLRT* Bootstrapped likelihood ratio test,

Best performing FMM (‘three-subgroup, seven-factor’) in font bold

^a^Log-likelihood was not replicated

^b^Model did not converge

### Factor mixture modelling

A direct comparison of all competing models demonstrated that the ‘three-subgroup/seven-factor’ FMM provided the best fit to the data and was superior to all other models (CFA, LCA and FMM) based on all goodness-of-fit criteria (AIC, BIC, adjusted-BIC; see Table 2). The likelihood ratio test for the ‘three-subgroup/seven-factor’ FMM model was also significant (*p* = .01), suggesting that deleting a subgroup resulted in a significantly worse fit of the model. The ‘three-subgroup/seven-factor’ FMM also had a far better BIC value than any of the three/four/five or six-subgroup LCA models (3045, 2789, 2591 and 2590 points lower), but with estimating fewer parameters, and had a better BIC value (107 points lower) than the best-fitting 7-factor solution, suggesting parsimony in the description of the structure of sensory features in ASD. On the basis of this FMM analysis in the current sample, heterogeneity in sensory features in ASD can therefore be best described by three more homogeneous sensory subgroups that differ in sensory severity gradients within and between groups along seven continuous factor scores.

### Class characterisation

Table 3 presents a description of the three sensory subgroups as derived from the FMM on different variables of interest. On average, subgroup 1 (*N* = 24; 7.1%; ‘sensory severe’) was characterised by more severe sensory features across all seven SSP domain scores compared to subgroup 2 (*N* = 51; 15.8%; ‘sensory moderate’) and subgroup 3 (*N* = 257; 77.1%; ‘sensory low’). Overall, estimated ES for group differences across SSP domain and total scores were large between subgroup 1 and subgroup 3 (*d* range 0.5–2.5), moderate between subgroup 2 and subgroup 3 (*d* range 0.4–1.5) and low/moderate between subgroup 1 and subgroup 2 (*d* range 0.2–2.5). The largest group differences were observed on ‘Movement sensitivity’ (*d* > 1.1), while the lowest differences were found for ‘Taste/Smell sensitivity’ (*d* < 0.5). Note that while subgroup 3 showed relatively less sensory impairment than subgroup 1 and 2, comparisons with typical reference samples still indicated either probable or definite sensory atypicalities across most of the SSP domains (Supplementary Materials).

**Table 3:** Sensory subgroup comparisons on key clinical variables of interest (*N* = 332)

	Mean (SD)			Effect size		
	Subgroup 1: severe (*N* = 24)	Subgroup 2: moderate (*N* = 51)	Subgroup 3: low (*N* = 257)	G1 vs. G2	G1 vs. G3	G2 vs. G3
Sex (males:females); % of females	8:16 (33%)	13:38 (25%)	74:183 (28%)	–	–	–
Age	16.0 (6.3)	14.3 (5.7)	16.3 (5.6)	0.30	− 0.06	− 0.35
Full-scale IQ	87.3 (17.9)	94.1 (22.8)	97.6 (20.6)	− 0.33	− 0.50	− 0.17
Tactile sensitivity	22.3 (6.6)	23.8 (6.9)	27.5 (5.5)	− 0.25	− 0.86	− 0.61
Taste/smell sensitivity	12.8 (5.3)	13.6 (5.2)	15.4 (4.9)	− 0.17	− 0.53	− 0.37
Movement sensitivity	4.9 (2.1)	8.5 (2.3)	13.5 (1.9)	− 1.05	− 2.52	− 1.47
Underresponsive/seeks sensation	21.1 (5.1)	23.6 (6.4)	27.0 (6.2)	− 0.38	− 0.92	− 0.53
Auditory filtering	13.1 (3.8)	14.3 (4.6)	17.8 (5.2)	− 0.22	− 0.89	− 0.67
Low energy/weak	17.6 (7.5)	19.3 (7.8)	24.4 (6.6)	− 0.24	− 0.94	− 0.70
Visual/auditory sensitivity	13.5 (3.1)	15.5 (4.9)	18.8 (4.9)	− 0.40	− 1.05	− 0.65
Total score	104.1 (21.0)	118.8 (23.5)	144.0 (24.5)	− 0.54	− 1.46	− 0.92
ADOS CSS social affect	6.7 (2.5)	6.9 (2.5)	6.3 (2.6)	− 0.07	0.14	0.21
ADOS CSS-RRB	5.2 (2.8)	5.1 (2.9)	4.8 (2.8)	0.03	0.14	0.11
SRS-2 SCI	82.2 (6.9)	79.4 (11.8)	71.8 (11.5)	0.24	0.88	0.65
RBS-R stereotyped	5.6 (4.9)	4.8 (4.2)	3.2 (3.6)	0.30	0.72	0.42
RBS-R self-injurious	2.1 (3.6)	2.0 (3.1)	1.3 (2.1)	0.02	0.34	0.32
RBS-R compulsive	4.1 (3.4)	3.0 (2.5)	2.3 (2.7)	0.39	0.64	0.25
RBS-R ritualistic/sameness	12.2 (8.1)	10.4 (7.1)	6.3 (5.3)	0.29	0.96	0.67
RBS-R restricted	3.6 (2.3)	2.9 (2.2)	2.4 (1.9)	0.36	0.61	0.25
Anxiety	2.7 (1.5)	2.7 (1.6)	2.5 (1.3)	− 0.02	0.16	0.18
Inattention	6.5 (2.3)	5.6 (2.4)	4.4 (3.2)	0.31	0.69	0.39
Hyperactivity/impulsivity	4.3 (3.2)	3.7 (2.9)	2.7 (2.8)	0.18	0.53	0.35
VABS socialisation	57.2 (15.43)	66.6 (17.7)	71.1 (15.9)	− 0.57	− 0.84	− 0.28
VABS daily living	61.6 (12.56)	70.1 (17.1)	73.8 (16.9)	− 0.51	− 0.72	− 0.22
VABS communication	68.8 (14.72)	75.3 (19.5)	75.3 (16.8)	− 0.38	− 0.38	0.00
VABS ABC	60.1 (12.62)	68.2 (16.1)	71.4 (14.7)	− 0.54	− 0.75	− 0.21

Effect size estimates = subgroup mean difference/overall SD, ES 0.2–0.3 = small, ES 0.5 = medium, ES > 0.8 = large, *RRB* Restricted and repetitive behaviours, *VABS* Vineland Adaptive Behaviour Scales-II, *ABC* Adaptive Behaviour Composite

**Table 4:** Multivariate multiple regression model predicting clinical variables by sensory class

	Sensory severe vs. low subgroup		Sensory moderate vs. low subgroup			
Variable	*b* SE (*b*)	*t*	95% CI	*b* SE (*b*)	*t*	95% CI
ADOS CSS-SA	− 0.06 (0.63)	− 0.10	[− 1.30, 1.18]	0.37 (0.49)	0.76	[− 0.60, 1.34]
ADOS CSS-RRB	− 0.10 (0.71)	− 0.14	[− 1.50, 1.30]	0.18 (0.56)	0.33	[− 0.91, 1.28]
**SRS-2 SCI**	**10.36 (2.68)**	3.86^*^	**[5.08, 15.64]**	**5.70 (2.10)**	2.72^*^	**[1.57, 9.84]**
**RBS-R stereotyped**	**2.35 (0.89)**	2.64^*^	**[0.59, 4.10]**	0.97 (0.70)	1.40	[− 0.40, 2.35]
RBS-R self-injurious	0.90 (0.64)	1.41	[− 0.36, 2.16]	0.99 (0.50)	1.98^+^	[0.01, 1.98]
**RBS-R compulsive**	**2.14 (0.67)**	3.18^*^	**[0.81, 3.46]**	0.35 (0.53)	0.67	[− 0.69, 1.39]
**RBS-R ritualistic/sameness**	**6.96 (1.38)**	5.04^*^	**[4.24, 9.68]**	**3.75 (1.08)**	3.47^*^	**[1.62, 5.88]**
RBS-R restricted	1.24 (0.50)	2.49^+^	[0.26, 2.23]	0.26 (0.39)	0.68	[− 0.50, 1.03]
DAWBA anxiety (linear)	0.43 (0.25)	1.74	[− 0.06, 0.91]	0.23 (0.19)	1.17	[− 0.15, 0.61]
**DAWBA anxiety (quadratic)**	0.24 (0.25)	0.98	[− 0.24, 0.72]	**0.67 (0.19)**	3.48^*^	**[0.29, 1.05]**
DAWBA anxiety (cubic)	− 0.21 (0.24)	− 0.91	[− 0.68, 0.25]	− 0.01 (0.18)	− 0.05	[− 0.37, 0.35]
**ADHD inattention**	**2.00 (0.73)**	2.74^*^	**[0.56, 3.45]**	0.85 (0.57)	1.49	[− 0.28, 1.98]
ADHD hyperactivity/impulsivity	1.68 (0.68)	2.49	[0.35, 3.01]	0.93 (0.53)	1.76	[− 0.11, 1.97]
**VABS socialisation**	− **11.98 (3.78)**	− 3.16^*^	**[**− **19.44,** − **4.52]**	− 5.16 (2.96)	− 1.74	[− 11.00, 0.67]
VABS daily living	− 9.54 (3.80)	− 2.51^+^	[− 17.04, − 2.05]	− 2.14 (2.98)	− 0.72	[− 8.01, 3.72]
VABS communication	− 3.81 (3.36)	− 1.13	[− 10.43, 2.81]	0.30 (2.63)	0.12	[− 4.88, 5.48]
VABS ABC	− 8.03 (3.13)	− 2.56^+^	[− 14.20, − 1.85]	− 2.01 (2.45)	− 0.82	[− 6.84, 2.82]

*b* regression coefficient, *SE (b)* standard error of regression coefficient, *t t* statistic, *95% CI* 95% confidence interval of regression coefficient, *ABC* Adaptive Behaviour Composite, *ADOS CSS-SA, RRB* Autism Diagnostic Observation Schedule Calibrated Severity Scores for Social Affect and Restricted and Repetitive Behaviours, *SRS-2* Social Responsiveness Scale–2, *RBS-R* Repetitive Behaviour Scale-Revised, *ADHD* DSM-5 ADHD rating scale

*p < .01

^+^*p* < .05

**Figure 1:**
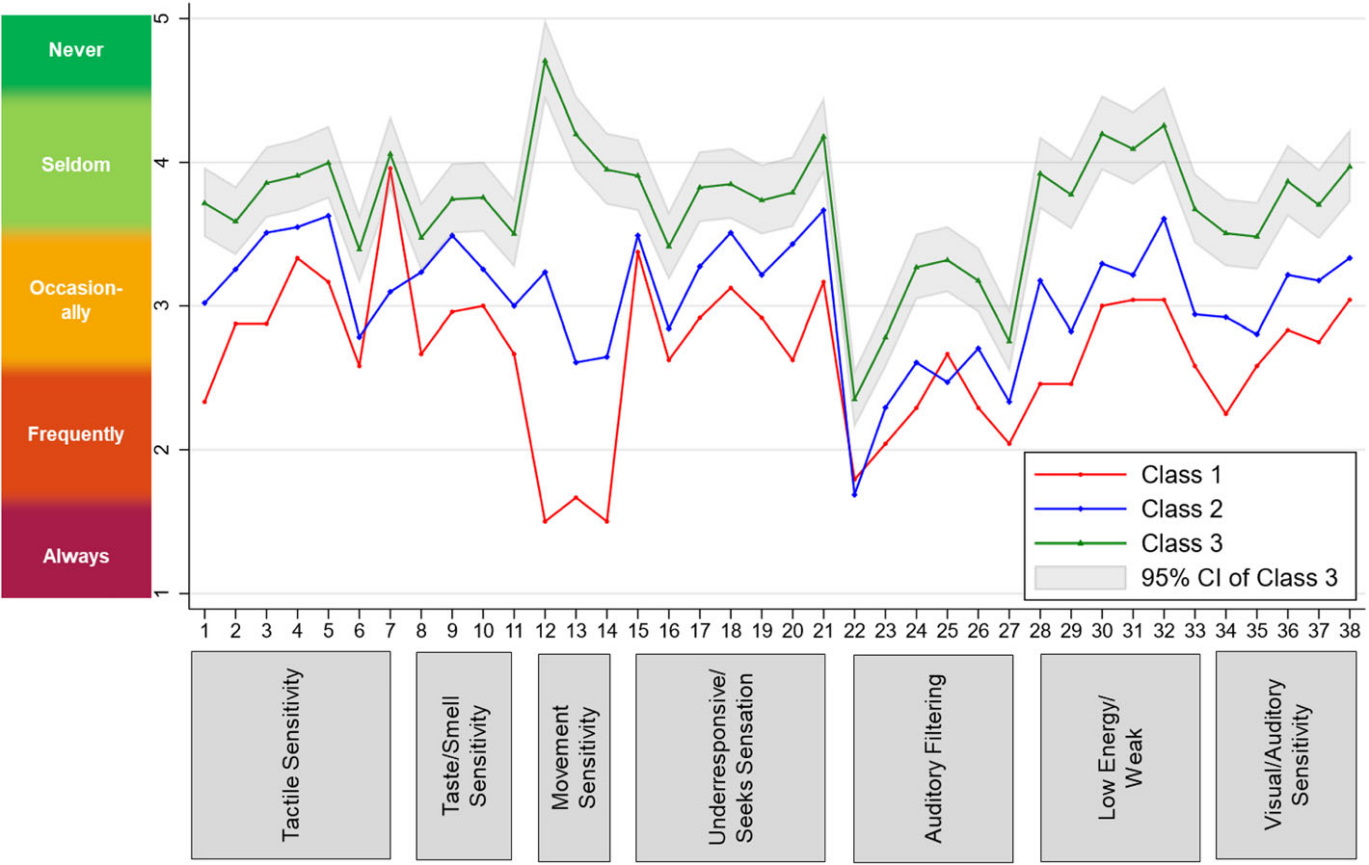
Three-subgroup, seven-factor FMM profile plot for the 38 items by class and associated SSP domain scores. Response options ranged from 1 = behaviour always present to 5 = behaviour never present

To evaluate whether sensory subgroups can be characterised by qualitative or quantitative differences, an item profile plot was created. As can be seen in Fig. 1, the response patterns on the 38 SSP items are very similar across the three subgroups and are mainly quantitatively ordered. Subgroup 3 tends to score ‘never’ or ‘seldom’ on most items, subgroup 2 on average endorses items with ‘occasionally’ and subgroup 1 scores ‘frequently’ or ‘always’. Strictly quantitative differences would be reflected in parallel response profiles on the 38 items, whereas qualitative differences would be reflected in a crossover between items and between subgroups. Crossovers in response profiles are largely absent in Fig. 1, with the exception for item 7 (“Rubs or scratches out a spot that has been touched”) and item 22 (“Is distracted or has trouble functioning if there is a lot of noise around”). While subgroup 1 scores lower (i.e. indicating more severe behaviour) on all other items compared to subgroup 2, subgroup 2 scores lower than subgroup 1 on items 7 and 22. Exploratory post hoc analyses suggested that group differences were significant for item 7 (*t*(45) = 2.03, *p* = .024), but not item 22 (*t*(45) = 0.48, *p* = .316).

### Subgroup interpretation

To aid in the interpretation of derived sensory subgroups, participants with ASD were compared to normative data on 1037 children without intellectual disabilities (ID) provided in the SSP manual (Supplementary Table 6). Subgroup 1, the sensory severe subgroup, showed definite differences across six of the seven SSP domains and probable differences for ‘Taste/Smell sensitivity’. Subgroup 2, characterised as the sensory moderate subgroup also demonstrated definite differences on the majority of the SSP domains, except for ‘Taste/Smell sensitivity’ and ‘Underresponsive/Seeks sensation’ (probable differences). In contrast to these two groups, subgroup 3 (‘sensory low’) showed definite differences only in ‘Auditory Filtering’ and probable/typical differences in four and three domains respectively. For the total sample, mean values on sensory domains fell either within the typical range (3 domains), probable range (3 domains) or definite range (1 domain: ‘Auditory Filtering’). This indicates that participants in the sample experienced sensory dysfunction to varying degrees across sensory modalities compared to a normative typically developing (TD) sample.

To further examine sensory features in the current ASD sample in relation to a typically developing group matched more closely in age (compared to the SSP normative data), additional data from the LEAP cohort was analysed on *N* = 132 TD individuals (Mean_age_ = 13.1, SD_age_ = 4.0, Range_age_ = 6.2–26.5; Mean_fsiq_ = 109.7, SD_fsiq_ = 12.8, Range_fsiq_ = 75.3–142.0). The TD sample has been described in more detail elsewhere [29]. Raw SSP scores of ASD participants were converted into standardised *z*-scores (mean = 0; *SD* = 1) relative to the TD sample and were calculated for each sensory class and SSP domain and total scores (Supplementary Table 7). Following Lane et al. [18], ‘Typical Performance’ relates to *z*-scores at or above − 1, ‘Probable Difference’ is indicated by *z*-scores between − 1 and − 2 (Supplementary Table 8). *Z*-scores that fall below − 2 are considered to indicate ‘Definite Difference’ and are likely to be functionally impairing [74]. Compared to the TD cohort, subgroup 1 showed definite differences across all sensory domains, and subgroup 2 showed definite differences in five of seven SSP domains. For subgroup 3, five of seven SSP domains indicated probable differences, while two SSP domains were classified as typical performance (movement sensitivity and low energy/weak). Thus overall, while there were relative differences in the severity of sensory features between sensory subgroups, even the low scoring subgroup exhibited on average sensory features that indicate atypical functioning.

### Sensory subgroup differences on clinical variables

Differences between sensory subgroups on key demographic variables ranged from small (age) to moderate (IQ) and were non-significant (*p* > .02). A cross-tabulation (chi-square) analysis found that there was also no significant difference in distribution by sex across subgroups (*x*^2^(2) = .509, *p* = .775).

Overall, individuals assigned to subgroup 1 (‘sensory severe’) had more severe behavioural symptoms (core and associated) and greater functional impairment followed by individuals in subgroup 2 (‘sensory moderate’), with individuals in subgroup 3 (‘sensory low’) having on average lower symptom scores and better adaptive functioning. Specifically, large effect size differences were found between subgroup 1 relative to subgroup 3 on social communication and interaction symptoms (SRS-2 SCI), ritualistic/sameness behaviours and adaptive functioning (VABS socialisation domain, VABS ABC), and moderate effect size differences were found for stereotyped behaviours, restricted behaviours, symptoms of inattention and hyperactivity/impulsivity and daily living skills. Effect size differences were small for symptoms of anxiety. Controlling for the effects of age and IQ, the MMR analysis broadly confirmed this pattern (Table 4). Compared to subgroup 3, individuals in subgroup 1 had significantly higher SRS-2 SCI scores (*p* < .001), more symptoms of inattention (*p* = .007) and greater adaptive functioning deficits, specifically for the socialisation domain (*p* = .002). In addition, individuals in the sensory severe subgroup had significantly higher scores on the RBS-R domains of stereotyped, compulsive and ritualistic/sameness behaviours (all *p*s < .009). Comparing subgroups 2 and 3, effect size differences were moderate for SRS-2 SCI and ritualistic/sameness behaviours, and small for all other variables, including anxiety symptoms. Based on the MMR results, individuals in subgroup 2 had significantly higher SRS-2 SCI scores (*p* = .007) and more severe ritualistic/sameness behaviours (*p* = .001). A significant quadratic effect for anxiety was observed (*p* = .001), suggesting that groups differed significantly only at higher, and not lower, levels of risk (i.e. only when the probability of an anxiety disorder exceeds 70%).

## Discussion

Sensory features are a frequently occurring and clinically impairing group of symptoms among individuals diagnosed with ASD. However, despite clinical significance, prominent heterogeneity in sensory features has stifled our understanding of this group of symptoms. The current study aimed to further our understanding of the heterogeneity in sensory features by utilising, for the first time in the literature, factor mixture modelling (FMM) to systematically compare dimensional, categorical and dimensional-categorical hybrid structures of sensory features in a large and well-characterised sample of individuals with ASD.

### Structure of sensory features

Results demonstrated that a multidimensional, i.e. hybrid factor model yielded the most parsimonious representation of sensory features in ASD, specifically a three-subgroup/seven-factor structural model. According to this model, individuals with ASD can be stratified into three more homogenous sensory subgroups, while allowing for heterogeneity in the severity of sensory features within these groups along seven specific continuous factor scores/domains. This suggests that neither dimensional-only nor categorical-only structural models can account sufficiently for the broad heterogeneity in sensory features observed in individuals with ASD.

The sensory-based subgroups we identified, interpreted as ‘sensory severe’, ‘sensory moderate’ and ‘sensory low’ showed statistically significant differences (see large effect sizes in Table 3) in overall sensory symptom severity, as well as across specific sensory domains, and in particular in movement sensitivity. The derived subgroups were characterised by a severity gradient rather than showing qualitative differences in sensory features, in line with some previous studies [12, 17]. The absence of specific sensory patterns across subgroups however contrasts with several other sensory-based subgrouping studies in ASD. For example, in a series of cluster analytic studies using the SSP in samples of children with ASD, Lane and colleagues [18, 19, 75, 76] identified four sensory subtypes: sensory adaptive, taste smell sensitive, postural inattentive and generalised sensory difference. Similarly, in a study that utilised the Sensory Experiences Questionnaire [77], four sensory subgroups were identified, two of which were mainly distinguished by a severity gradient (‘Mild’ and ‘Extreme-Mixed’) and two showing qualitative differences (‘Sensitive-Distressed Subtype’, ‘Attenuated-Preoccupied’, [16]). A potential reason for these discrepancies may relate to the choice of analytical approaches. In FMM, variability in item scores is both modelled by categorical and continuous latent factors, while in cluster analysis and/or latent profile/class analysis (LPA, LCA), all variability between participants is assumed to be captured by categorical subgroups [39]. This leads to LCA extracting more subgroups to account for inter-individual variability, while in FMM, fewer subgroups are required to explain variation and covariation among the test items by permitting within-group covariance structures [78]. Our data supports this conclusion, as the four- to six-class LCA models were found to have a better fit to the data than the three-subgroup LCA model. Alternatively, the different nature of the samples studied may have also affected the findings. More specifically, previous studies by Lane et al. and Ausderau focused on toddlers and younger children, while the sample used for our investigation spanned a wider age range from children to adults. It has been suggested that over time, the more specific patterns of sensory features identified in toddlers and younger children tend to restructure according to a sensory severity continuum [12]. Although this suggestion is tentative and warrants further investigation using longitudinal designs, a study that utilised SSP in older children and adolescents has, similarly to our study, identified three subgroups that differed in terms of overall severity of sensory features.

### Association with clinical characteristics of sensory subgroups

The sensory subgroups showed statistically significant differences in their associations with the severity of core and co-occurring symptoms, and level of adaptive functioning after accounting for the potential confounding effects of age and IQ. This provides suggestive support for the potential clinical utility of the identified three subgroups. More specifically, participants in the severe compared to the low sensory group had more severe social-communicative symptoms, greater deficits in adaptive social functioning skills, more symptoms of inattention and more restricted and repetitive behaviours, in particular stereotyped, compulsive and ritualistic/sameness behaviours. Compared to the low sensory group, individuals in the moderate sensory group had significantly greater social-communication difficulties, more ritualistic/sameness behaviours and greater symptoms of anxiety at higher, but not lower levels of risk (i.e. only when the probability of an anxiety disorder exceeded 70%).

These results are in line with findings from studies that utilised both variable- and person-centred approaches. For instance, in a sample of children with ASD aged between 6 and 10 years, Hilton et al. [10] found that higher severity of sensory features, measured by the full Sensory Profile, was associated with higher severity of social-communication symptoms, assessed by the SRS-2. In addition, a subtyping study by Ausderau et al. [79] found that two of the subgroups characterised by the highest severity of sensory problems showed the most impairments in the communication and socialisation domains of the Vineland Adaptive Behaviour Scale-II. While the causal relationship between sensory features and social-communication challenges in ASD is not established, it may be the case that sensory features may result in the individual withdrawing from social-communicative environments that are over stimulating, thereby further restricting opportunities for social learning. Conversely, the link between restricted and repetitive behaviours, particularly stereotypes, compulsions and rituals/sameness behaviours, and sensory features have been highlighted by several studies [7–9, 80], and RRBs may serve as a self-regulatory function in situations of high arousal [81]. There is also increasing evidence on the association between atypical sensory features and anxiety [7, 11], including two sensory-based subtyping studies that have identified that sensory severe subgroups showed more severe anxiety symptoms in both toddlers [17] and older children and adolescents [12]. Although the exact mechanisms underpinning the relationship between anxiety and atypical sensory features remain to be clarified, it has been suggested that due to heightened responses to sensory stimuli, individuals characterised by atypical sensory processing experience their environment as threatening and unpredictable, which in turn leads to increased levels of anxiety [7]. It has to be noted however that the effect size differences between the sensory severe/moderate group and sensory mild group found in the current sample were low (*d* = 0.23 and *d*’ = 0.09 respectively). A lack of significant difference in anxiety symptoms between the severe and low group in the current study may be a result of the limited sample size in the sensory severe group. In summary, the current findings highlight the importance of comprehensively investing these related phenotypes in future studies and stress the need for understanding causality.

### Research and clinical implications

To better understand the complex issue of ASD heterogeneity, it will now be important to examine the three derived sensory subgroups across different research areas: (a) developmental trajectories and stability of sensory subgroups over time, (b) response to intervention, (c) behavioural and clinical factors that associate with subgroups and (d) neurobiological and genetic mechanisms related to subgroups.

For example, it is possible that individuals from the different sensory subgroups might follow different developmental trajectories, which could be helpful in determining prognosis and identifying developmental opportunities for targeted interventions. When implemented within longitudinal designs, subgroups with distinct sensory profiles can serve as indicators of later outcomes, not only in relation to the categorical diagnostic outcome status but also the presence of other clinical features. In this context, it will also be important to assess the stability of sensory subgroups over time. Individuals in these sensory subgroups may also respond differently to different treatment options. Finally, one could hypothesise that individuals from the same sensory subgroup may converge on similar etiological pathways and thus may respond more similarly to treatment approaches [82]. In this context, it will be critical to identify biological and genetic markers that capture diversity in sensory features in ASD. Although it is clear that the timing and magnitude of responses to sensory inputs are different and can have detrimental effects in individuals with ASD, the genetic and neurobiological underpinnings are currently poorly understood. For example, based on currently available findings, it is unclear whether the atypical sensory features in ASD are a consequence of impairments in bottom-up [83] or top-down processing [84–86] or the impairments of both levels of processing [87]. These inconsistencies can be attributed to the fact that previous studies have utilised small samples (*N* < 25) which most likely included individuals belonging to different sensory-based subgroups. The importance of pre-selecting individuals based on their sensory profiles is illustrated by a study conducted by Green et al. [87] that showed that individuals with ASD with and without sensory hypersensitivity could be distinguished based on the profile of amygdala reactivity and amygdala-orbitofrontal cortex coupling during the presentation of aversive sensory stimuli. However, although innovative, this study only considered sensory hypersensitivity rather than comprehensive sensory functioning profiles. Therefore, the identified subgroups have the potential to advance our understanding of the neurobiology of atypical sensory features in ASD and the next important step in this research programme is to characterise potential neurobiological and genetic differences among individuals belonging to distinct sensory-based subgroups that we have reported here.

Relating the sensory subgroups to typically developing (TD) data from both the normative SSP standardisation sample and a closely age- and IQ-matched TD comparison group recruited as part of the LEAP cohort suggested that subgroups differed in the level of clinical relevance of their sensory features. While the ‘sensory low’ group had reduced sensory features in comparison to the other subgroups, compared to the TD reference data, even this subgroup showed on average sensory features that indicate atypical functioning across most domains assessed by the SSP. Individuals in the ‘sensory severe’ and ‘sensory moderate’ group experienced significant difficulties across most sensory domains, as indicated by the high frequency of ‘Definite difference’ or ‘Probable difference’ classifications across sensory domains. For individuals in these groups, the severity of sensory features experienced is likely functionally limiting [26] and indicates a clinical concern. In fact, participants classified in these clusters meet criteria for clinical cases of sensory processing disorder as described by Lane et al. [18]. Thus, if validated in future studies, these subtypes may offer a means during diagnostic evaluation to identify those individuals with clinically relevant levels of sensory features that require additional support and potentially benefit most from sensory-based therapies.

## Limitations

Several limitations of the study have to be noted. First, the results were derived using a single parent-report measure of sensory features, the SSP, and are necessarily influenced by the item content of the measure. More specifically, despite their clinical importance, the SSP provides only limited coverage of sensory hypo-sensitivity and unusual sensory interest [22]. It is therefore possible that by relying on the SSP, which does not align well with the DSM-5 subtypology of sensory symptoms, our study was not able to afford a more fine-grained characterisation of the sensory subgroups. Thus, while the severity gradient of the identified sensory subgroups speaks to their clinical utility, the reliance on a single measure and limited coverage of relevant sensory domains in DSM-5 warrants additional and complimentary work. Adding to this, by relying on a single parent-report measure, the results may reflect the measure-specific construct(s) rather than sensory structures in autistic individuals more generally. It will therefore be of crucial importance for future studies to utilise multiple measures that provide comprehensive sampling of all key sensory domains and drawing on different measurement formats (e.g. parent-report, self-report, observation) in order to derive content- and method-independent sensory-based subgroups [88]. In this context, it will be critical to test in a confirmatory setting (i.e. in a hypothesis-driven manner) the predictive utility of the present results in a larger and independent ASD sample.

Second, although identified subgroups were associated with several key symptom and functional domains, therefore suggesting potential clinical utility, it is important to highlight that due to the cross-sectional design of the study, these findings are necessarily preliminary. It will be important to further explore the predictive validity of the identified subgroups within a longitudinal study. As additional data on this longitudinal sample becomes available, we will evaluate these questions in more detail. Third, the FMM imposes a common factor structure in each subgroup and thus does not allow to test for different factor structures in different latent subgroups (e.g. testing for measurement invariance across subgroups). The size of the current sample, although being larger than in most previous studies, did not allow us to address this question.

## Conclusions

Heterogeneity within the autism spectrum is, perhaps, the biggest challenge to basic and clinical research and translation of research into clinical practice [5]. By applying for the first time factor mixture modelling in the context of sensory features in ASD, we demonstrated that a multidimensional hybrid model combining dimensional and categorical latent factors provided the most parsimonious representation of sensory features in ASD. This approach has the potential to enable a more fine-grained understanding of heterogeneity in sensory features in ASD and may be crucial to advance future clinical, genetic and neurobiological research.

## Supplementary information

**Additional file 1: **Supplementary Materials. **Table 1.** SSP items and sub-scale assignment. **Table 2.** Model comparisons. **Table 3.** Item endorsements, standardised factor loadings, and item R^2^ values for 7-factor model. **Table 4.** 7-factor correlations. **Table 5.** Bifactor model: Standardised factor loadings and explained common item variance for general factor and uncorrelated specific factors. **Table 6.** Classification per SSP manual (based on normative data from 1,037 children without ID). **Table 7.** Z-scores relative to typically developing comparison sample (*N*=132). **Table 8.** Classification per Z-scoring. **Figure 1.** Information curves for individual subscales and for all items as a function of theta. **Figure 2.** Reliability curves for individual subscales and for all items as a function of theta.

## Data Availability

The datasets generated and/or analysed during the current study are not publicly available due to an embargo period but are available from the corresponding author on reasonable request.

## Abbreviations

AASP: Adolescent/Adult Sensory Profile
ADHD: Attention-deficit/hyperactivity disorder
ADOS: Autism Diagnostic Observation Schedule
ASD: Autism spectrum disorder
CFA: Confirmatory factor analysis
CSS: Calibrated severity scores
DAWBA: Development and Well-Being Assessment
DSM-5: Diagnostic and Statistical Manual of Mental Disorders, 5th Edition
DSM-IV: Diagnostic and Statistical Manual of Mental Disorders, 4th Edition
DSM-IV-TR: Diagnostic and Statistical Manual of Mental Disorders, 4th Edition text-revision
EU-AIMS: European Autism Interventions—A Multicentre Study for Developing New Medications
FA: Factor analysis
FMM: Factor mixture modelling
ICD-10: International Statistical Classification of Diseases and Related Health Problems, 10th Revision
ITSP: Infant Toddler Sensory Profile
IQ: Intelligence quotient
LCA: Latent class analysis
LEAP: Longitudinal European Autism Project
RBS-R: Repetitive Behaviour Scale-Revised
RRB: Restricted and repetitive behaviours
SA: Social affect
SBQ: Sensory Behaviour Questionnaire
SEQ: Sensory Experiences Questionnaire
SP: Sensory Profile
SRS-2: Social Responsiveness Scale, 2nd Edition
SSP: Short Sensory Profile
TD: Typically developing
VABS: Vineland Adaptive Behaviour Scale-II
WAIS-III/IV: Wechsler Adult Intelligence Scale–Third Edition/Fourth Edition
WASI-II: Wechsler Abbreviated Scales of Intelligence–Second Edition
WISC-III/IV: Wechsler Intelligence Scale for Children–Third Edition/Fourth Edition
