# Supplementary material for: Dissecting the phenotypic heterogeneity in sensory features in autism spectrum disorder: a factor mixture modelling approach
Source: Mol Autism. 2020 Aug 31;11:67. doi: 10.1186/s13229-020-00367-w (PMC7457751; doi:10.1186/s13229-020-00367-w)
Supplement: Supplementary file 1 — Additional file 1: Supplementary Materials. Table 1. SSP items and sub-scale assignment. Table 2. Model comparisons. Table 3. Item endorsements, standardised factor loadings, and item R2 values for 7-factor model. Table 4. 7-factor correlations. Table 5. Bifactor model: Standardised factor loadings and explained common item variance for general factor and uncorrelated specific factors. Table 6. Classification per SSP manual (based on normative data from 1,037 children without ID). Table 7. Z-scores relative to typically developing comparison sample (N=132). Table 8. Classification per Z-scoring. Figure 1. Information curves for individual subscales and for all items as a function of theta. Figure 2. Reliability curves for individual subscales and for all items as a function of theta. [file 13229_2020_367_MOESM1_ESM.docx]

**Supplementary Materials**

*Results – Confirmatory Factor Analysis*

Three separate CFAs were run to test different factor structures of the 38 items of the SSP: the original 7-factor solution (4), a 6-factor solution (5), and a novel 5-factor solution that has been partially proposed in previous studies (6, 7), but has never been psychometrically assessed. Supplementary Table 1 provides an item-by-item comparison of the factor models tested. In contrast to the 7-factor solution, the 6-factor solution splits items from the ‘Underresponsive/Seeks Sensation’ and ‘Auditory Filtering’ sub-scale into items relating to ‘Underresponsive/Seeks Sensation’ (items 15-18, 27), ‘Hyposensitivities’ (item 19, 21, 23, 26) and ‘Visual/Auditory Sensitivity’ (items 20, 22, 24, 25). In addition, items loading on ‘Tactile Sensitivity’ and ‘Movement Sensitivity’ were combined to form a ‘Tactile Sensitivity and Movement’ sub-scale. The 5-factor solution collapses the original sub-scales for ‘Tactile Sensitivity’, ‘Movement Sensitivity’ and ‘Visual/Auditory Sensitivity’ into a ‘Hyper-reactivity’ sub-scale while retaining ‘Taste/Smell Sensitivity’, ‘Underresponsive/Seeks Sensation’, ‘Auditory Filtering’ and ‘Low Energy/Weak’. Previous studies also included items from ‘Taste/Smell Sensitivity’ in a ‘Over-responsivity’ construct (6, 7), yet based on internal discussions and prior reports suggesting that items largely reflect food selectivity rather than hyper-reactivity/over-responsivity to food items (8), we decided to model this domain separately.

To account for the ordinal structure of the SSP item-data (Likert-scale: scores can range from 0=always to 5=never), the confirmatory factor analysis (CFA) was re-run in an Item Response Theory (IRT)/Item Factor Analysis (IFA) framework using weighted least squares estimation (WLSMV) and theta parameterization based on the polychoric correlation matrices using a diagonal weight matrix with standard errors and mean- and variance adjusted chi-square test statistics. Three separate CFAs were run to test the model fit of the original 7-factor solution (4), the 6-factor solution (5) and the novel 5-factor solution. In all CFAs, factor means and factor variances were set to 0 and 1 respectively for model identification.

Several fit indices to evaluate goodness of model fit were computed: The Comparative Fit Index (CFI), the Tucker Lewis Index (TLI), and the Root Mean Square Error of Approximation (RMSEA) and its 90% confidence interval (90% CI; MacCallum, Browne, & Sugawara, 1996). A CFI and TLI >0.90 indicates adequate fit (>0.95 for excellent fit). An RMSEA <0.08 (90% CI <0.08) indicates adequate fit (<0.05 for good fit; Browne and Cudeck, 1992), with smaller values indicating better fit. Multiple indices were used since they provide different information about model fit (CFI/TLI: absolute fit; RMSEA: fit adjusted for model parsimony). Used together, these indices provide a more conservative and reliable evaluation of the underlying factor solution (Jaccard & Wan, 1996).

*Global model fit*

Supplementary Table 2 provides a detailed summary of all CFA models tested. The 5-factor solution produced adequate model fit (CFI=0.931; TLI=0.926; RMSEA=0.071), while the 6-factor solution had adequate to good model fit (CFI=0.946; TLI=0.942; RMSEA=0.063). The 7-factor solution produced the best overall fit to the data, with good to excellent model fit across multiple fit indices (CFI=0.958; TLI=0.954; RMSEA=0.055).

Item endorsements, standardised factor loadings, and item R^2^ for the best-fitting 7-factor solution are summarised in Supplementary Table 3. Item endorsements, defined as any rating lower than 5 (i.e. rating 5 = when presented with the opportunity, subject never responds in this manner), demonstrated some variability, with the lowest endorsement for item 12 (33%; ‘Becomes anxious or distressed when feet leave the ground’) and highest for item 22 (95%; ‘Is distracted or has trouble functioning if there is a lot of noise around’). Overall, standardised factor loadings were large and significant within each factor (λ ≥ .52, *p*<.001), suggesting that all items are measuring the hypothesised construct they load on. The lowest factor loadings were observed for item 3 (λ=0.58; ‘Avoids going barefoot’) and item 4 (λ=0.52; ‘Reacts emotionally or aggressively to touch’). This pattern was also present in the item R^2^ values reflecting the proportion of variance accounted for by the factor, which ranged from 27% to 94%. For the 7-factor solution, correlations among factor scores ranged from low/moderate between ‘Low Energy/Weak (LOW)’ and ‘Taste/Smell sensitivity (TAS)’ to high between ‘Tactile Sensitivity (TAC)’ and ‘Visual/Auditory sensitivity (VAS)’ (see Supplementary Table 4), suggesting that the factors measure separate but related constructs.

*Local model fit*

To further evaluate the 7-factor solution, sources of local misfit were identified by inspecting standardised residual correlations between items loading on the same factor, where larger negative residuals indicate that items are less related than predicted by the model. Subdomains for ‘Taste/Smell sensitivity’, ‘Movement sensitivity’, and ‘Low Energy/Weak’ showed little evidence of local misfit (maximum negative residual: -0.072). In contrast, on ‘Tactile Sensitivity’ item 6 vs. item 1 (-0.185) and item 7 vs. item 1 (-0.158) showed relatively greater misfit. On ‘Underresponsive/seeks sensation’, items 16, 17, and 18 had greater negative residuals with item 21 (-0.146, -0.159, -0.158 respectively). For ‘Auditory Filtering’, item 22 vs. item 26 (-0.192) exhibited the largest misfit, followed by item 23 vs. item 24 (-0.173). On ‘Visual/Auditory Sensitivity’, item 34, 35 vs. item 38 (-0.152, -0.139 respectively) showed relatively greater misfit. Interestingly, the comparisons producing the largest local misfits, i.e. item 6 (‘Has difficulty standing in line or close to other people’) vs. item 1 (‘Expresses distress during grooming’) and item 22 (‘Is distracted or has trouble functioning if there is a lot of noise around’) vs. item 26 (‘Doesn't respond when name is called but you know the child's hearing is OK’), include items tapping into socially-relevant behaviours (item 6 and item 26) rather than specific non-social sensory behaviours in tactile and auditory domains.

*Test information & reliability*

To evaluate how reliable each of the seven SSP subscales are in measuring across different levels of a persons’ trait/factor (in IRT this is referred to as theta), test information and reliability was computed by collapsing information across relevant items and plotted for different levels of theta (Supplementary Figure 1, 2). This feature of IRT thus allows to go beyond asking how reliable or precise a score in general is, and instead helps to evaluate how precise a measurement is for individuals in high, medium, and low trait ranges. Test information can be transformed into reliability (information/(information+1), with test information values of above 4 corresponds to a reliability coefficient of 0.80 and information value of 10 reflecting reliability of 0.90. Inspection of information curves suggest that each individual SSP subscale is more reliable at measuring around a mean trait level than at either a low/severe or high/mild end of a person’s sensory traits as evidenced by pronounced peaks around theta=0. However, subscales differed in reliability, particularly around a mean trait level, with ‘Taste/Smell Sensitivity’, ‘Movement Sensitivity’, and ‘Low Energy/Weak’ having higher reliability than all other subscales. ‘Tactile Sensitivity’ demonstrated the lowest reliability overall compared to all other sub-scales, with only acceptable reliability in the theta range between -1 and 0. Interestingly, ‘Auditory Filtering’ was the only subscale that demonstrated good reliability across a broader range of the trait, and particularly at higher levels of the trait (theta range with information >=4: -1.6 to +1.6) compared to all other subscales. Evaluating the SSP scale as a whole, i.e. pooling test information across all 38 items, it appears that the SSP has excellent reliability around the mean trait level and good reliability at the low/severe trait level. However, it sharply tapers off towards the high/mild extreme end.

*Hierarchical bifactor model*

Following an in-depth evaluation of the 7-factor measurement model, we further considered the applicability of a bifactor structural model. In the bifactor model, each item was allowed to load on both a general factor that is assumed to underlie all items, as well as to load on one of the seven specific factors previously identified. While the general factor accounts for common variance among all scale items, specific factors capture item covariation that is independent of the covariation due to the general factor (9). The general factor thus represents a conceptually broad construct (i.e. sensory behaviours across domains), while the specific factors relate to conceptually more narrow constructs of interest or sub-domains (e.g. ‘Tactile Sensitivity’). Identical to the 7-Factor correlated model, the bifactor model produced excellent model fit (CFI=0.959; TLI=0.954; RMSEA=0.056), albeit being less parsimonious by requiring the estimation of more parameters. Supplementary Table 5 shows standardised item loadings, as well as the percentage of item variance accounted for by the general and specific factors. All factor loadings on the specific factors remained significant (λ ≥ .17, *p*<.001), yet as expected were noticeably smaller (average difference in factor loadings: λ^diff^ = -.24), since items were now allowed to also load on the general factor. All items loaded significantly on the general factor (λ ≥ .37, *p*<.001), suggesting that all items contribute to a broad sensory construct. However, there was wide variability across items in the magnitude of factor loadings on the general vs. specific factor. For ‘Taste/Smell Sensitivity’ and Low Energy/Weak’, all items displayed stronger factor loadings on their designated specific factors compared to the general factor, while all items for ‘Visual/Auditory Sensitivity’ had stronger factor loadings on the general sensory factor. A mixed pattern of findings was observed for ‘Tactile Sensitivity’, ‘Movement Sensitivity’, and ‘Underresponsive/Seeks Sensation’, with most items loading higher on the specific than the general factor. Overall, this suggests the presence of multidimensionality, i.e. multi-faceted sensory traits that share specific item content, alongside a common latent sensory trait that allows scaling individuals along a single common dimension.

**Supplementary Table 1** SSP items and sub-scale assignment

| Item  No. | SSP item description | 5F | 6F | 7F |
| --- | --- | --- | --- | --- |
| 1 | Expresses distress during grooming | HYP | TMS | TAC |
| 2 | Prefers long-sleeved clothing when it is warm or short sleeves when it is cold | HYP | TMS | TAC |
| 3 | Avoids going barefoot | HYP | TMS | TAC |
| 4 | Reacts emotionally or aggressively to touch | HYP | TMS | TAC |
| 5 | Withdraws from splashing water | HYP | TMS | TAC |
| 6 | Has difficulty standing in line or close to other people | HYP | TMS | TAC |
| 7 | Rubs or scratches out a spot that has been touched | HYP | TMS | TAC |
| 8 | Avoids certain tastes or food smells | TAS | TAS | TAS |
| 9 | Will only eat certain tastes | TAS | TAS | TAS |
| 10 | Limits self to particular food textures/temperatures | TAS | TAS | TAS |
| 11 | Picky eater, especially regarding food textures | TAS | TAS | TAS |
| 12 | Becomes anxious or distressed when feet leave the ground | HYP | TSM | MOV |
| 13 | Fears falling or heights | HYP | TSM | MOV |
| 14 | Dislikes activities where head is upside down | HYP | TSM | MOV |
| 15 | Enjoys strange noises/seeks to make noise for noise's sake | UND | SSD | UND |
| 16 | Seeks all kinds of movement and this interferes with daily routines | UND | SSD | UND |
| 17 | Becomes overly excitable during movement activity | UND | SSD | UND |
| 18 | Touches people and objects | UND | SSD | UND |
| 19 | Doesn't seem to notice when face or hands are messy | UND | HYPO | UND |
| 20 | Jumps from one activity to another so that it interferes with play | UND | VAS | UND |
| 21 | Leaves clothing twisted on body | UND | HYPO | UND |
| 22 | Is distracted or has trouble functioning if there is a lot of noise around | AUD | VAS | AUD |
| 23 | Appears to not hear what you say | AUD | HYPO | AUD |
| 24 | Can't work with background noise | AUD | VAS | AUD |
| 25 | Has trouble completing tasks when the radio is on | AUD | VAS | AUD |
| 26 | Doesn't respond when name is called but you know the child's hearing is OK | AUD | HYPO | AUD |
| 27 | Has difficulty paying attention | AUD | SSD | AUD |
| 28 | Seems to have weak muscles | LOW | LOW | LOW |
| 29 | Tires easily, especially when standing or holding particular body position | LOW | LOW | LOW |
| 30 | Has a weak grasp | LOW | LOW | LOW |
| 31 | Can't lift heavy objects | LOW | LOW | LOW |
| 32 | Props to support self (even during activity) | LOW | LOW | LOW |
| 33 | Poor endurance/tires easily | LOW | LOW | LOW |
| 34 | Responds negatively to unexpected or loud noises | HYP | AVS | VAS |
| 35 | Holds hands over ears to protect ears from sound | HYP | AVS | VAS |
| 36 | Is bothered by bright lights after others have adapted to the light | HYP | AVS | VAS |
| 37 | Watches everyone when they move around the room | HYP | AVS | VAS |
| 38 | Covers eyes or squints to protect eyes from light | HYP | AVS | VAS |

Note:
5F = 5-factor model: HYP = Hyper-reactivity; TAS = Taste & Smell sensitivity; UND = Underresponsive/Seeks sensation; AUD = Auditory Filtering; LOW = Low Energy/Weak
6F = 6-factor model (Tomchek et al. 2014): TMS = Tactile & Movement sensitivity, TAS = Taste & Smell sensitivity; SSD = Sensory seaking/distractibility; HYPO = Hypo-responsivity; ; LOW = Low Energy/Weak; AVS = Auditory & Visual sensitivity

7F = 7-factor model (McIntosh et al. 1999): TAC = Tactile sensitivity; TAS = Taste & Smell sensitivity; MOV = Movement sensitivity; UND = Underresponsive/Seeks sensation; AUD = Auditory Filtering; LOW = Low Energy/Weak; VAS = Visual/Auditory Sensitivity

**Supplementary Table 2** Model comparisons

| SSP Model tested | χ^2^ | Par | CFI | TLI | RMSEA (90% CI) |
| --- | --- | --- | --- | --- | --- |
| Unidimensional | 4953.34** | 190 | 0.727 | 0.711 | 0.139 (0.136-0.143) |
| 5F | 1737.28** | 200 | 0.931 | 0.926 | 0.071 (0.067-0.075) |
| 6F | 1496.03** | 205 | 0.946 | 0.942 | 0.063 (0.058-0.067) |
| 7F | 1299.14** | 211 | 0.958 | 0.954 | 0.055 (0.051-0.060) |
| Bi-factor 7F | 1277.29** | 228 | 0.959 | 0.954 | 0.056 (0.051-0.060) |

SSP = Short Sensory Profile; Unidimensional = Single common factor model; 5F = novel 5-factor model; 6F = 6-factor model from Tomchek et al. (2014);7F = Original 7-factor model from McIntosh et al. (1999); Par = Estimated number of parameters; CFI = Confirmatory Fit Index; TLI = Tucker-Lewis Index; RMSEA = root mean square error of approximation; WRMR = weighted root mean square residual; CI = confidence interval

*** *p* < 0.0001

**Supplementary Table 3** Item endorsements, standardised factor loadings, and item R^2^ values for 7-factor model

| Factor | Item  No. | SSP item description | Endorsements  (in %) | Standardised factor loading | Item R^2^ |
| --- | --- | --- | --- | --- | --- |
| TAC | 1 | Expresses distress during grooming | 58 | 0.782 | 0.611 |
|  | 2 | Prefers long-sleeved clothing when it is warm or short sleeves when it is cold | 62 | 0.610 | 0.372 |
|  | 3 | Avoids going barefoot | 54 | 0.583 | 0.340 |
|  | 4 | Reacts emotionally or aggressively to touch | 61 | 0.522 | 0.272 |
|  | 5 | Withdraws from splashing water | 52 | 0.603 | 0.363 |
|  | 6 | Has difficulty standing in line or close to other people | 75 | 0.700 | 0.491 |
|  | 7 | Rubs or scratches out a spot that has been touched | 44 | 0.667 | 0.444 |
| TAS | 8 | Avoids certain tastes or food smells | 63 | 0.911 | 0.830 |
|  | 9 | Will only eat certain tastes | 54 | 0.910 | 0.827 |
|  | 10 | Limits self to particular food textures/temperatures | 54 | 0.949 | 0.900 |
|  | 11 | Picky eater, especially regarding food textures | 67 | 0.877 | 0.769 |
| MOV | 12 | Becomes anxious or distressed when feet leave the ground | 33 | 0.969 | 0.938 |
|  | 13 | Fears falling or heights | 57 | 0.863 | 0.745 |
|  | 14 | Dislikes activities where head is upside down | 52 | 0.826 | 0.683 |
| UND | 15 | Enjoys strange noises/seeks to make noise for noise's sake | 55 | 0.625 | 0.390 |
|  | 16 | Seeks all kinds of movement and this interferes with daily routines | 71 | 0.833 | 0.693 |
|  | 17 | Becomes overly excitable during movement activity | 59 | 0.837 | 0.701 |
|  | 18 | Touches people and objects | 59 | 0.734 | 0.539 |
|  | 19 | Doesn't seem to notice when face or hands are messy | 59 | 0.651 | 0.423 |
|  | 20 | Jumps from one activity to another so that it interferes with play | 66 | 0.784 | 0.615 |
|  | 21 | Leaves clothing twisted on body | 48 | 0.659 | 0.434 |
| AUD | 22 | Is distracted or has trouble functioning if there is a lot of noise around | 95 | 0.821 | 0.674 |
|  | 23 | Appears to not hear what you say | 93 | 0.834 | 0.695 |
|  | 24 | Can't work with background noise | 85 | 0.781 | 0.610 |
|  | 25 | Has trouble completing tasks when the radio is on | 80 | 0.757 | 0.573 |
|  | 26 | Doesn't respond when name is called but you know the child's hearing is OK | 83 | 0.783 | 0.614 |
|  | 27 | Has difficulty paying attention | 93 | 0.736 | 0.541 |
| LOW | 28 | Seems to have weak muscles | 50 | 0.945 | 0.892 |
|  | 29 | Tires easily, especially when standing or holding particular body position | 60 | 0.949 | 0.901 |
|  | 30 | Has a weak grasp | 43 | 0.932 | 0.868 |
|  | 31 | Can't lift heavy objects | 44 | 0.891 | 0.793 |
|  | 32 | Props to support self (even during activity) | 40 | 0.872 | 0.760 |
|  | 33 | Poor endurance/tires easily | 62 | 0.889 | 0.791 |
| VAS | 34 | Responds negatively to unexpected or loud noises | 68 | 0.774 | 0.599 |
|  | 35 | Holds hands over ears to protect ears from sound | 75 | 0.829 | 0.687 |
|  | 36 | Is bothered by bright lights after others have adapted to the light | 61 | 0.882 | 0.777 |
|  | 37 | Watches everyone when they move around the room | 68 | 0.675 | 0.456 |
|  | 38 | Covers eyes or squints to protect eyes from light | 60 | 0.867 | 0.751 |

Note: TAC = Tactile sensitivity; TAS = Taste & Smell sensitivity; MOV = Movement sensitivity; UND = Underresponsive/Seeks sensation; AUD = Auditory Filtering; LOW = Low Energy/Weak; VAS = Visual/Auditory Sensitivity

**Supplementary Table 4** 7-factor correlations

| Factor | TAC | TAS | MOV | UND | AUD | LOW | VAS |
| --- | --- | --- | --- | --- | --- | --- | --- |
| TAC | - |  |  |  |  |  |  |
| TAS | .52 | - |  |  |  |  |  |
| MOV | .64 | .43 | - |  |  |  |  |
| UND | .53 | .35 | .39 | - |  |  |  |
| AUD | .57 | .37 | .42 | .69 | - |  |  |
| LOW | .50 | .33 | .50 | .36 | .41 | - |  |
| VAS | .70 | .40 | .50 | .60 | .67 | .37 | - |

Note: TAC = Tactile sensitivity; TAS = Taste & Smell sensitivity; MOV = Movement sensitivity; UND = Underresponsive/Seeks sensation; AUD = Auditory Filtering; LOW = Low Energy/Weak; VAS = Visual/Auditory Sensitivity

**Supplementary Table 5** Bifactor model: Standardised factor loadings and explained common item variance for general factor and uncorrelated specific factors

| Item  No. | General | TAC | TAS | MOV | UND | AUD | LOW | VAS |
| --- | --- | --- | --- | --- | --- | --- | --- | --- |
| 1 | 0.681 | 0.170 |  |  |  |  |  |  |
| 2 | 0.510 | 0.333 |  |  |  |  |  |  |
| 3 | 0.453 | 0.465 |  |  |  |  |  |  |
| 4 | 0.371 | 0.667 |  |  |  |  |  |  |
| 5 | 0.465 | 0.515 |  |  |  |  |  |  |
| 6 | 0.610 | 0.201 |  |  |  |  |  |  |
| 7 | 0.556 | 0.357 |  |  |  |  |  |  |
| 8 | 0.565 |  | 0.669 |  |  |  |  |  |
| 9 | 0.452 |  | 0.811 |  |  |  |  |  |
| 10 | 0.508 |  | 0.792 |  |  |  |  |  |
| 11 | 0.424 |  | 0.799 |  |  |  |  |  |
| 12 | 0.671 |  |  | 0.656 |  |  |  |  |
| 13 | 0.579 |  |  | 0.647 |  |  |  |  |
| 14 | 0.526 |  |  | 0.686 |  |  |  |  |
| 15 | 0.453 |  |  |  | 0.429 |  |  |  |
| 16 | 0.595 |  |  |  | 0.595 |  |  |  |
| 17 | 0.576 |  |  |  | 0.672 |  |  |  |
| 18 | 0.546 |  |  |  | 0.466 |  |  |  |
| 19 | 0.479 |  |  |  | 0.438 |  |  |  |
| 20 | 0.601 |  |  |  | 0.451 |  |  |  |
| 21 | 0.505 |  |  |  | 0.379 |  |  |  |
| 22 | 0.691 |  |  |  |  | 0.362 |  |  |
| 23 | 0.673 |  |  |  |  | 0.471 |  |  |
| 24 | 0.590 |  |  |  |  | 0.570 |  |  |
| 25 | 0.567 |  |  |  |  | 0.561 |  |  |
| 26 | 0.621 |  |  |  |  | 0.486 |  |  |
| 27 | 0.596 |  |  |  |  | 0.395 |  |  |
| 28 | 0.482 |  |  |  |  |  | 0.821 |  |
| 29 | 0.545 |  |  |  |  |  | 0.772 |  |
| 30 | 0.479 |  |  |  |  |  | 0.805 |  |
| 31 | 0.459 |  |  |  |  |  | 0.769 |  |
| 32 | 0.532 |  |  |  |  |  | 0.678 |  |
| 33 | 0.505 |  |  |  |  |  | 0.728 |  |
| 34 | 0.598 |  |  |  |  |  |  | 0.544 |
| 35 | 0.655 |  |  |  |  |  |  | 0.515 |
| 36 | 0.706 |  |  |  |  |  |  | 0.531 |
| 37 | 0.566 |  |  |  |  |  |  | 0.287 |
| 38 | 0.698 |  |  |  |  |  |  | 0.505 |
| Expl.  variance | 50% | 6% | 7% | 5% | 8% | 7% | 11% | 6% |

Note: General = General factor solution; TAC = Tactile sensitivity; TAS = Taste & Smell sensitivity; MOV = Movement sensitivity; UND = Underresponsive/Seeks sensation; AUD = Auditory Filtering; LOW = Low Energy/Weak; VAS = Visual/Auditory Sensitivity; Expl. Variance = Explained common item variance in %


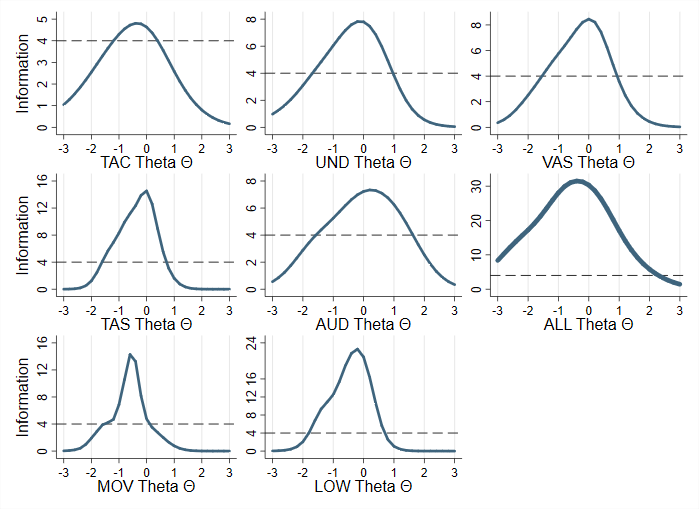


**Supplementary Figure 1** Information curves for individual subscales and for all items as a function of theta; Dashed line: information=4, relates to a reliability of .80; TAC = Tactile sensitivity; TAS = Taste & Smell sensitivity; MOV = Movement sensitivity; UND = Underresponsive/Seeks sensation; AUD = Auditory Filtering; LOW = Low Energy/Weak; VAS = Visual/Auditory Sensitivity; ALL = Information summed across all items


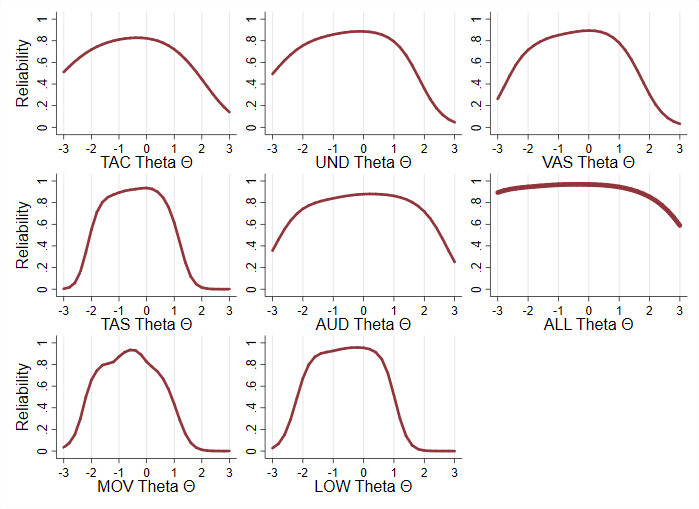


**Supplementary Figure 2** Reliability curves for individual subscales and for all items as a function of theta; TAC = Tactile sensitivity; TAS = Taste & Smell sensitivity; MOV = Movement sensitivity; UND = Underresponsive/Seeks sensation; AUD = Auditory Filtering; LOW = Low Energy/Weak; VAS = Visual/Auditory Sensitivity; ALL = Reliability summed across all items

**Supplementary Table 6** Classification per SSP manual (based on normative data from 1,037 children without ID)

|  | **Classification** | | |  | **Total sample** (N=332) | |
| --- | --- | --- | --- | --- | --- | --- |
|  | Subgroup 1: Severe (*N*=24) | Subgroup 2: Moderate (N=51) | Subgroup 3: Low (N=257) |  | Mean (SD) | Classification |
| Tactile Sensitivity | Definite | Definite | Probable |  | 26.97 (6.24) | Probable |
| Taste/Smell Sensitivity | Probable | Probable | Typical |  | 15.15 (4.92) | Typical |
| Movement Sensitivity | Definite | Definite | Probable |  | 12.53 (3.24) | Typical |
| Underresponsive/Seeks Sensation | Definite | Probable | Typical |  | 27.82 (5.94) | Typical |
| Auditory Filtering | Definite | Definite | Definite |  | 17.22 (5.42) | Definite |
| Low Energy/Weak | Definite | Definite | Probable |  | 23.67 (7.07) | Probable |
| Visual/Auditory Sensitivity | Definite | Definite | Typical |  | 17.93 (5.07) | Probable |
| SSP Total score | Definite | Definite | Probable |  | 142.86 (26.62) | Probable |

**Supplementary Table 7** Z-scores relative to typically developing comparison sample (*N*=132)

|  | **Classification** | | |  |
| --- | --- | --- | --- | --- |
|  | Subgroup 1: Severe (*N*=24) | Subgroup 2: Moderate (N=51) | Subgroup 3: Low (N=257) |  |
| Tactile Sensitivity | -2.83 | -2.42 | -1.44 |  |
| Taste/Smell Sensitivity | -2.48 | -2.14 | -1.38 |  |
| Movement Sensitivity | -4.63 | -2.82 | -0.30 |  |
| Underresponsive/Seeks Sensation | -2.45 | -1.89 | -1.11 |  |
| Auditory Filtering | -2.36 | -2.12 | -1.39 |  |
| Low Energy/Weak | -2.23 | -1.90 | -0.76 |  |
| Visual/Auditory Sensitivity | -3.48 | -2.76 | -1.59 |  |
| SSP Total score | -4.12 | -3.27 | -1.81 |  |

**Supplementary Table 8** Classification per Z-scoring

|  | **Classification** | | |  |
| --- | --- | --- | --- | --- |
|  | Subgroup 1: Severe (*N*=24) | Subgroup 2: Moderate (N=51) | Subgroup 3: Low (N=257) |  |
| Tactile Sensitivity | Definite | Definite | Probable |  |
| Taste/Smell Sensitivity | Definite | Definite | Probable |  |
| Movement Sensitivity | Definite | Definite | Typical |  |
| Underresponsive/Seeks Sensation | Definite | Probable | Probable |  |
| Auditory Filtering | Definite | Definite | Probable |  |
| Low Energy/Weak | Definite | Probable | Typical |  |
| Visual/Auditory Sensitivity | Definite | Definite | Probable |  |
| SSP Total score | Definite | Definite | Probable |  |

Key: Z-scores at or above −1 are classified as “Typical Performance.”; Z-scores that fall between −1 and −2 are classified as “Probable Difference.”; Z-scores that fall below −2 are considered to indicate “Definite Difference”

**Supplementary References**

1. Charman T, Loth E, Tillmann J, Crawley D, Wooldridge C, Goyard D, et al. The EU-AIMS Longitudinal European Autism Project (LEAP): clinical characterisation. Molecular Autism. 2017;8(1):27.

2. Lane AE, Molloy CA, Bishop SL. Classification of Children With A utism S pectrum D isorder by Sensory Subtype: A Case for Sensory‐Based Phenotypes. Autism Research. 2014;7(3):322-33.

3. McIntosh D, Miller L, Shyu V. Development and validation of the short sensory profil. In: Dunn W, editor. Sensory profile: User’s manual (pp. San Antonio: The Psychological Corporation; 1999. p. 59-73.

4. McIntosh D, Miller L, Shyu V, Dunn W. Overview of the short sensory profile (SSP). The sensory profile: Examiner’s manual. 1999:59-73.

5. Tomchek SD, Huebner RA, Dunn W. Patterns of sensory processing in children with an autism spectrum disorder. Research in Autism Spectrum Disorders. 2014;8(9):1214-24.

6. Wigham S, Rodgers J, South M, McConachie H, Freeston M. The interplay between sensory processing abnormalities, intolerance of uncertainty, anxiety and restricted and repetitive behaviours in autism spectrum disorder. Journal of Autism and Developmental Disorders. 2015;45(4):943-52.

7. Mazurek MO, Vasa RA, Kalb LG, Kanne SM, Rosenberg D, Keefer A, et al. Anxiety, sensory over-responsivity, and gastrointestinal problems in children with autism spectrum disorders. Journal of abnormal child psychology. 2013;41(1):165-76.

8. Williams ZJ, Failla MD, Gotham KO, Woynaroski TG, Cascio C. Psychometric evaluation of the short sensory profile in youth with autism spectrum disorder. Journal of autism and developmental disorders. 2018;48(12):4231-49.

9. Reise SP. The rediscovery of bifactor measurement models. Multivariate behavioral research. 2012;47(5):667-96.
